# Dopaminergic Neurons from Midbrain-Specified Human Embryonic Stem Cell-Derived Neural Stem Cells Engrafted in a Monkey Model of Parkinson’s Disease

**DOI:** 10.1371/journal.pone.0041120

**Published:** 2012-07-17

**Authors:** Marcel M. Daadi, Brad A. Grueter, Robert C. Malenka, D. Eugene Redmond, Gary K. Steinberg

**Affiliations:** 1 Department of Neurosurgery, Stanford School of Medicine, Stanford, California, United States of America; 2 Stanford Institute for Neuro-Innovation and Translational Neurosciences, Stanford School of Medicine, Stanford, California, United States of America; 3 Molecular Medicine Research Institute, Sunnyvale, California, United States of America; 4 Psychiatry and Behavioral Sciences, Stanford School of Medicine, Stanford, California, United States of America; 5 Departments of Psychiatry and Neurosurgery, Yale University School of Medicine, New Haven, Connecticut, United States of America; University of South Florida, United States of America

## Abstract

The use of human embryonic stem cells (hESCs) to repair diseased or injured brain is promising technology with significant humanitarian, societal and economic impact. Parkinson’s disease (PD) is a neurological disorder characterized by the loss of midbrain dopaminergic (DA) neurons. The generation of this cell type will fulfill a currently unmet therapeutic need. We report on the isolation and perpetuation of a midbrain-specified self-renewable human neural stem cell line (hNSCs) from hESCs. These hNSCs grew as a monolayer and uniformly expressed the neural precursor markers nestin, vimentin and a radial glial phenotype. We describe a process to direct the differentiation of these hNSCs towards the DA lineage. Glial conditioned media acted synergistically with fibroblastic growth factor and leukemia inhibitory factor to induce the expression of the DA marker, tyrosine hydroxylase (TH), in the hNSC progeny. The glial-derived neurotrophic factor did not fully mimic the effects of conditioned media. The hNSCs expressed the midbrain-specific transcription factors Nurr1 and Pitx3. The inductive effects did not modify the level of the glutamic acid decarboxylase (GAD) transcript, a marker for GABAergic neurons, while the TH transcript increased 10-fold. Immunocytochemical analysis demonstrated that the TH-expressing cells did not co-localize with GAD. The transplantation of these DA-induced hNSCs into the non-human primate MPTP model of PD demonstrated that the cells maintain their DA-induced phenotype, extend neurite outgrowths and express synaptic markers.

## Introduction

Parkinson’s disease (PD) is a neurodegenerative disorder characterized by the loss of dopaminergic (DA) neurons in the substantia nigra compacta, resulting in decreased DA input to the caudate and putamen. Symptoms include tremor, rigidity, bradykinesia and instability. Neural transplantation is a promising strategy to improve dopaminergic dysfunction in PD. Over 25 years of research using fetal mesencephalic tissue, as a source of DA neurons, has demonstrated the therapeutic potential of cell transplantation therapy in rodents [1], [2] and non-human primate (NHP) animal models [3] and in human patients [4], [5]. However, the use of fetal tissue in the transplantation procedure is compromised by numerous problems, including limited availability, high tissue variability that translates into inconsistent functional outcome, and resulting dyskinesias [6], [7]. Stem cells offer an alternative source of DA neurons. The main characteristic that makes human embryonic stem cells (hESCs) an attractive source of cells for clinical use is their ability to generate, under controlled conditions, virtually an unlimited number of progeny with potential to differentiate into a functionally specialized group of neurons. However, the derivation of homogenous, self-renewable hNSCs from hESCs and the identification of signals that regulate their fate are still prevalent challenges for this translational research.

We have previously demonstrated that in addition to a mitogenic effect, the basic fibroblast growth factor (bFGF) treatment of neurospheres-astroglia co-culture, induces tyrosine hydroxylase (TH, a marker for DA neurons) expression and other new neurotransmitter phenotypes in the forebrain-derived neurospheres [8]. Activin and bone morphogenetic protein-2 (BMP2), two transforming growth factor-β (TGFβ)-related growth factors, were identified as potential glial determinants responsible for the TH induction [9]. However, these known factors were not competent to induce TH expression in the stem cell-derived progeny [8]. Yet, glial-derived soluble factors acted in synergy with bFGF to instruct the forebrain-derived stem cell progeny to express the DA phenotype [8].

Pluripotent hESCs are a promising source of neural cells to study the biology of cellular diversity and for cellular therapy. Based on early studies using mouse ESCs [10], [11], the derivation of dopaminergic neurons from hESCs have been achieved by exposure to FGF8, sonic hedgehog (SHH), small molecules, such as CHIR [12] activators of the Wnt signaling pathway and co-culture with feeder cell layers, such as PA6 or amniotic membrane matrix, or through the derivation of floor plate precursors [12]–[25].

Neural induction from ESC cultures is the predominant default pathway and occurs in the absence of instructive signals [26]. However, under these conditions the induction is not purely neural and cannot be efficiently maintained in vitro. Reliable protocols have been developed to enrich for neural lineages from hESCs [see review [27]]. This process consists of directly inducing neural lineage elaboration from pluripotent hESCs, followed by a short-term perpetuation of the neural precursors to generate neural progeny. The neural precursors may be induced from hESCs through: 1) embryoid body (EB) formation through aggregation of undifferentiated hESCs in suspension cultures [28], [29], 2) co-culture of hESCs with a feeder layer, such as stromal cell lines MS5, PA6 or S17 [13], [30], 3) inhibition of the TGFβ signaling to allow the default neural induction pathway to take place [31]–[34] or 4) exposure of the hESC to HepG2 conditioned medium or neural inducing media [35], [36].

Growth factor-responsive and self-renewable multipotent hNSCs have been isolated from hESCs [37]–[39]. We have previously reported that these hNSCs generate a large number of nestin, vimentin and 3CB2-expressing progeny, do not express the pluripotency markers SSEA4, OCT4 or Nanog and do not form tumors in nude or normal rats [37]. The hNSCs exhibited stable growth and differentiated into the three principal cell types: neurons, astrocytes and oligodendrocytes. In the present study, we report the induction of the dopaminergic phenotype in this self-renewable multipotent hNSCs. We also expand on our previous finding on DA-inducing conditions for murine NSCs [8] and report that glial soluble factor(s) acted synergistically with bFGF and LIF to specifically induce TH expression in the hNSC progeny. For the first time, we report that DA neurons generated under these conditions maintain the DA phenotype in the methyl-4-phenyl-1,2,3,6-tetrahydropyridine (MPTP)-monkey model of PD.

## Results

### Isolation, Perpetuation and Characterization of the hNSCs

**Figure 1:**
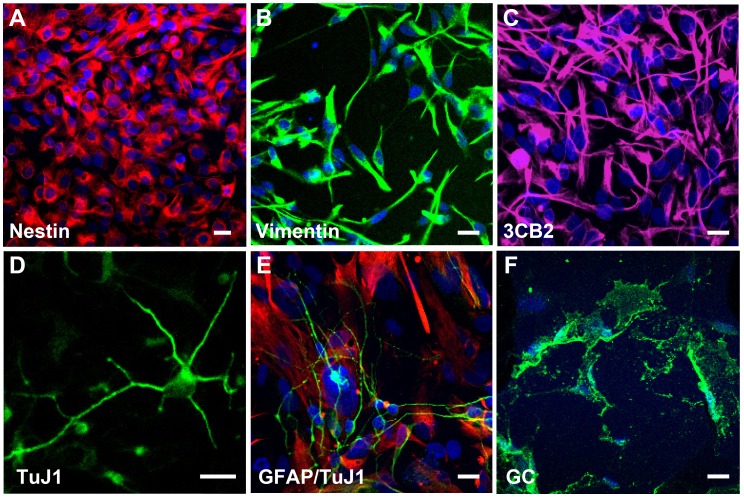
The hNSCs uniformly express markers of neural stem cells. The isolated hNSCs were passaged every 5–7 days, as described in the Methods section. They grew as a stable homogenous monolayer and uniformly expressed molecular features of NSCs. The top 3 panels: A, B, C represent photomicrographs of undifferentiated hNSCs immunostained with the neural precursor markers nestin (red, A) and vimentin (green, B) and the radial glial marker 3CB2 (purple, C). The cultures were counterstained with the nuclear DNA marker 4′,6-diamidine-2′-phenylindole dihydrochloride (DAPI, blue). The bottom panels (D, E, F) show 7-day old differentiated hNSC cultures processed for indirect immunocytochemistry. Differentiated hNSCs expressed the neuronal-specific marker TuJ1 (green D, E), the astroglia marker GFAP (red, E), the oligodendroglial marker GC (green, F) and DAPI, blue nuclei in all panels. Cell nuclei were stained with live cell marker DAPI (blue, F, G). Scale bars are 20 µm (A–F).

The hNSCs were isolated from hESCs and expanded, as we previously reported [37], in a defined culture medium, as detailed in Materials and Methods section. These culture conditions yielded stable cell lines that were consistently passaged every 5 to 7 days in vitro (DIV), grew as an adherent monolayer (Fig. 1) and expressed nestin, vimentin and the radial glial marker 3CB2 (Fig. 1 A–C). RT-PCR analysis confirmed that these hNSCs did not express the pluripotency transcripts Oct-4 and Nanog or the transcripts brachyury and foxa2, markers for mesoderm and endoderm respectively (data not shown) [37]. Upon removal of the mitogenic factors and plating on a coverslip pre-coated with poly-L-ornithine (PO) substrate, the hNSCs stopped proliferating and spontaneously differentiated into neurons, astrocytes and oligodendrocytes (Fig. 1 D–F). After 10 DIV, 33.4±3.3% of the total population were nestin+, 56.4±3.7% expressed the neuronal marker TuJ1, 9.8±1.6% expressed the astrocyte marker GFAP and 6.9±1.0% differentiated into galactocerebrocide positive (GC+) oligodendrocyte (Fig. 1 D–F).

### Induction of the Dopaminergic Phenotype in hNSC Progeny

**Figure 2:**
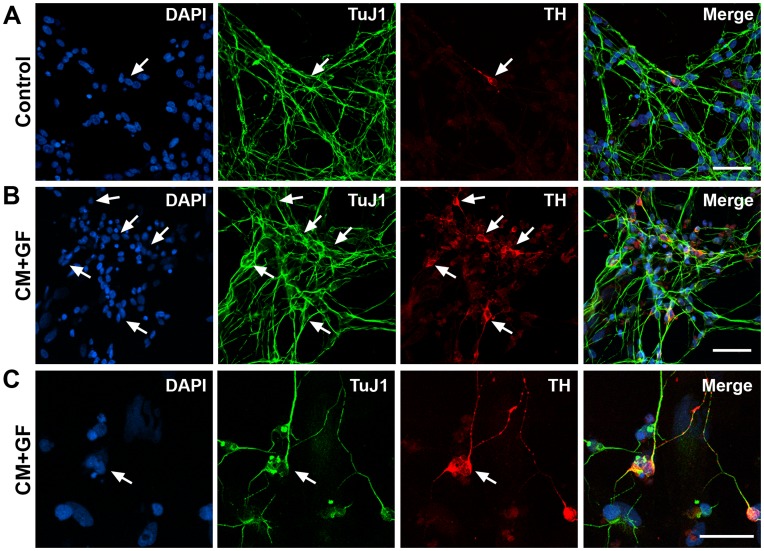
Treatment with the glial conditioned media (CM) and growth factors (GF) induce TH expression in hNSCs derived from hESCs. hNSCs were single cell dissociated in a defined medium and plated on poly-L-ornithine-coated glass coverslips either under control conditions (A) or in glial cell line conditioned media supplemented with the growth factors bFGF (20 ng/ml) and LIF 10 ng/ml for 10 days (B & C). Fixed cultures were stained with nuclear marker DAPI (blue) and processed for TH (red) and for the neuronal marker TuJ1 (green) immunocytochemistry, as described in Materials and Methods. Scale bars are 50 µm (A–C).

**Figure 3:**
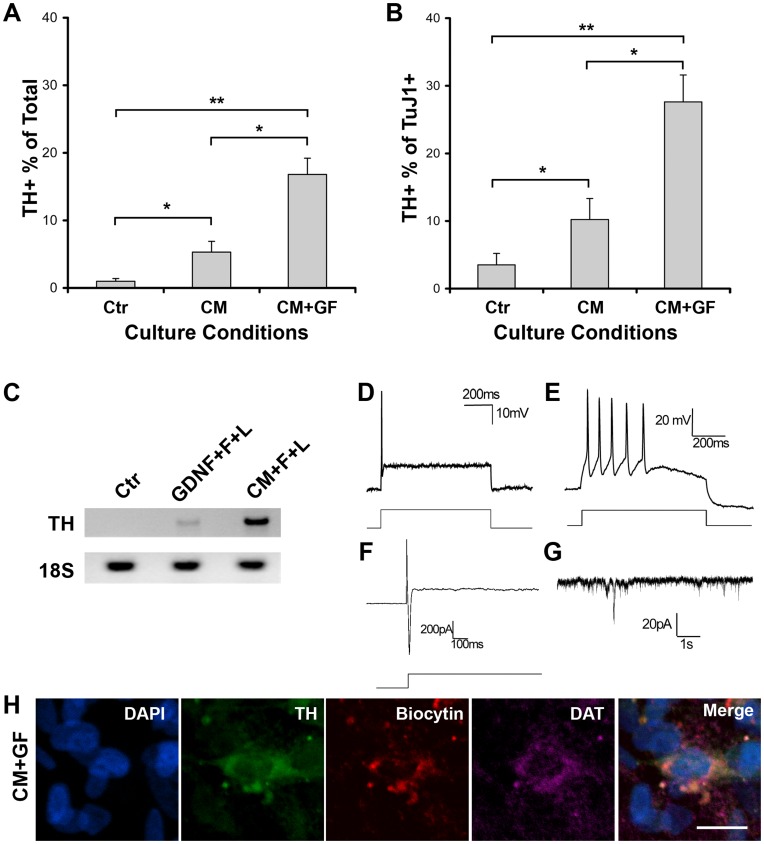
Electrophysiological properties of DA induced neurons. Quantitative analysis of the TH-inductive effects (**A** & **B**). The TH-IR neurons were expressed as a percentage of total live cells determined by DAPI staining and also as a percentage of the total number of TuJ1-IR neurons. Results are mean ± S.E.M. of experiments performed three times on independent culture preparations, each performed in duplicate. The TH induction with CM+F+L treatment produced significantly more TH+ cells compared to CM treatment alone (*P<0.01) and to non-treated control (**P<0.001). (**C**) The relative abundance of TH and 18 S transcripts was assessed by RT-PCR (see Materials and Methods section for the description of the primers used and the PCR conditions) in control (Ctr), GDNF+bFGF (F)+LIF (L) and in CM+F+L treated cultures (line +) which showed a strong induction of TH in CM+F+L culture condition. (**D**) Representative current clamp trace of action potentials elicited in response to a 400 pA current injection from a neuron. (**E**) Example of action potential burst in response to 400 pA current injections. (**F**) Voltage-activated sodium and potassium currents elicited in response to a −50 mV step. **(G)** Sample trace of spontaneous EPSCs in a neuron held at −70 mV. (**H**) Triple labeling immunocytochemical process of hNSC culture used for electrophysiological recording showing the co-localization of TH with biocytin and DAT. Scale bar is 20 µm (H).

To induce the dopaminergic phenotype, hNSCs were single cell dissociated, formulated in control medium at 0.5×10^6^ cell/ml and plated at a density of 2.5×10^5^ cell/cm^2^ on PO-coated glass coverslips in 24-well culture dishes. After a 2-hour culture period, the control media was replaced by TH-inducing media containing the growth factors (GF) bFGF at 10 ng/ml, LIF at 10 ng/ml and 75% (v/v) of the glial CM. The cultures were incubated and investigated for the TH induction within a period of 10 DIV (Fig. 2). In each culture, the neuronal and DA lineage species were determined using immunocytochemistry for TuJ1 and TH, respectively (Fig. 2 A–C). These cultures demonstrated induction of TH expression the TuJ1+ neuronal population (Fig. 2C) and a significant increase of the TH-IR cells by over 10 fold in cultures compared to control (Fig. 3A–B, *p<0.01, **p<0.001). After 10 days in culture, 16.8±2.4% of the total cells expressed TH, which represented approximately 27.6±4.0% of the total number of TuJ1-IR neurons (Fig. 3A, B). The CM alone increased by 2-fold the number of TH-expressing neurons. The addition of EGF to culture medium did not alter the number of TH-expressing cells (data not shown) while the omission of bFGF and LIF significantly mitigated the inductive effect (Fig. 3A, B, *p<0.01, **p<0.001). Since glial CM contain glial-derived neurotrophic factor (GDNF), a neurotrophic factor for the midbrain DA neuron [40], we asked the question whether GDNF may be involved in the induction of the dopaminergic phenotype. The treatment of cultures with saturating concentrations of GDNF (100 ng/ml) in the presence of FGF2 and LIF slightly stimulated the expression of the TH gene expression but did not fully mimic the glial conditioned media (Fig. 3C). Thus, a novel and more potent factor mediates the inductive effects.

### Electrophysiological Properties of the DA-induced Neurons

To investigate the electrophysiological properties of the DA neurons, whole cell voltage and current clamp recordings were made from DA-induced hNSC cultures. Injection of a depolarizing current triggered a single action potential in 3 of 9 hNSC-differentiated progeny (Fig. 3D) with bursting occurring in one cell (Fig. 3E). Voltage-activated sodium and potassium currents were elicited from 4 of 9 cells in response to a voltage step (Fig. 3F). Spontaneous excitatory post-synaptic currents (sEPSCs) were observed in 4 of 9 cells (Fig. 3G). While recording, the dye biocytin was injected into the recorded cell and immunocytochemical analysis confirmed the DA identity of the recorded cells (Fig. 3H). The TH-induced cells co-localized the dopamine transporter DAT (Fig. 3H).

### Midbrain Molecular Identity of the hNSCs

**Figure 4:**
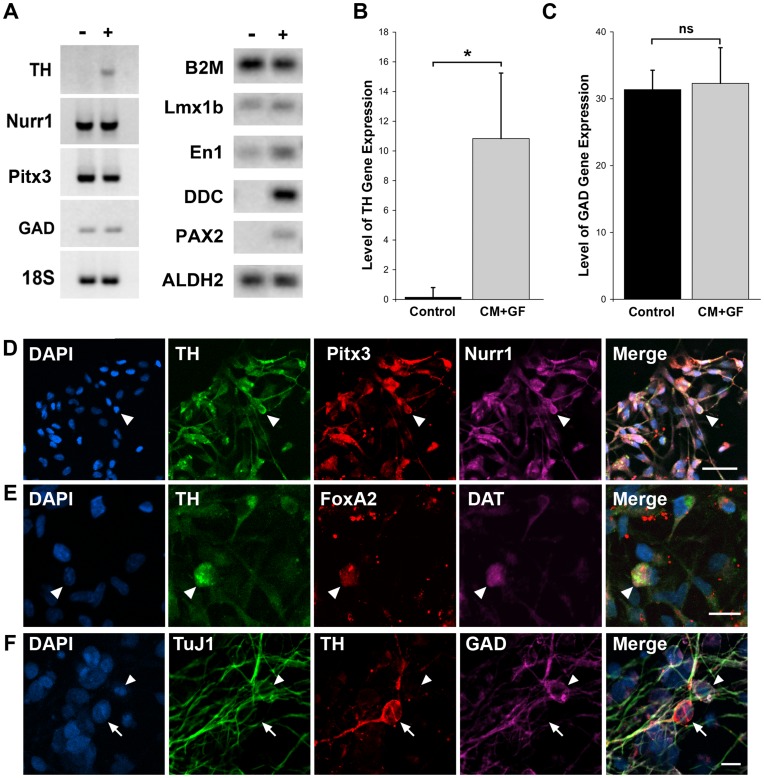
CM + GF stimulate TH and midbrain gene expression but not GAD. (**A**): Total RNA samples were extracted from hNSCs cultured for 10 days in the absence (line −) or presence (line +) of CM + bFGF + LIF. The relative abundance of TH, Nurr1, Pitx3, GAD and 18 S was assessed by RT-PCR (see Materials Methods section for the description of the primers used and the PCR conditions). CM+GF treated cultures (line +) showed a selective increase in PAX2, En1, TH, and DDC transcripts. (**B, C**): Gel densitometry software (Image J) was used to quantify amplified PCR products in 3 independent experiments. TH mRNA was significantly increased in CM+GF treated culture in comparison to control (* p<0.01), while GAD mRNA expression did not show significant differences between control and CM+GF treated cultures (ns  =  not significant). CM+GF treated cultures were immunostained for TH/Pitx3/Nurr1 (**D**) and TH/FoxA2/DAT (E). Photomicrographs in (**F**) show example of distinct cells expressing either the GABAergic maker GAD (purple, arrow head) or the DA marker TH (red, arrow). Scale bars are 50 µm (D), 20 µm (E), 10 µm (F).

The specification and maintenance of the midbrain DA fate during development is controlled by the nuclear receptor transcription factor, Nurr1, and the homeobox transcription factor Pitx3 [reviewed in [41]]. To determine whether or not the induction of the DA phenotype in the hES-derived hNSCs was due to the acquisition of a true midbrain DA identity, we looked for the expression of the A9 midbrain DA-specific transcription factors. Control and CM + GF-treated cultures where harvested after 10 DIV and investigated for the presence of TH, Nurr1, Pitx3, Lmx1b, En1, Pax2, DDC, GAD and ALDH2 transcripts. Semi-quantitative and Taq-man quantitative RT-PCR analyses revealed that both control and DA-induced cultures expressed *Nurr1* and *Pitx3* genes (Fig. 4A), while Pax2, En1 and DDC were clearly up-regulated in the DA-induced cultures. Immunocytochemistry for TH and the midbrain DA-specific markers Pitx3, Nurr1 and FoxA2 (Fig. 4D–E) confirmed the appropriate midbrain identity of the TH+ neurons.

### The DA-induced Neurons do not Co-localize GABA

We have previously shown that murine forebrain-derived DA neurons co-express the GABAergic phenotype, a default neurotransmitter phenotype for the neural stem cell progeny [8]. To investigate whether DA-inducing conditions alter the expression of the enzyme glutamic acid decarboxylase (GAD) marker for neurons expressing the neurotransmitter GABA or if the DA-induced neurons co-express GAD, we looked for the expression level of GAD transcripts in control and in CM+GF treated cultures. Our data demonstrate that the level of GAD expression was similar in both control and treated cultures (Fig. 4A, C) suggesting that the inducing effects were specific to the *TH* gene (Fig. 4A, B). To determine whether or not the TH-expressing cells co-localize GABA, we performed triple immunocytochemistry for TuJ1, TH and GAD. The data show that the TH did not co-localize with GABA in the same cells (Fig. 4F). Together these findings demonstrated that the hESC-derived DA neurons differ from the forebrain-derived NSCs and that the DA-induced phenotype in the hESC-derived NSC progeny possess traits of the midbrain DA neurons.

### Engraftment into MPTP-lesioned NHP

**Figure 5:**
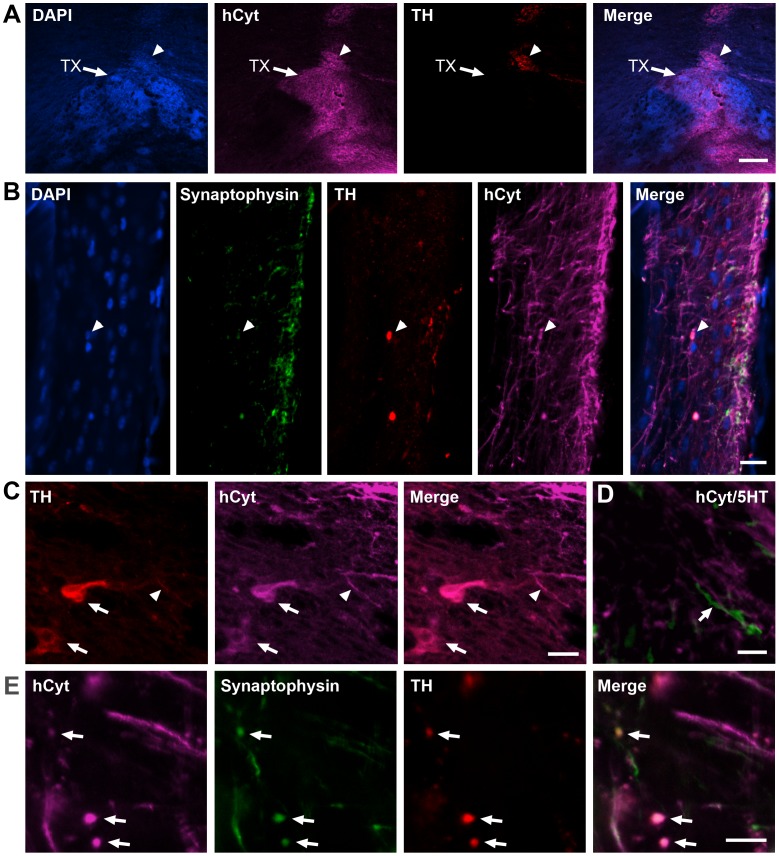
Grafted dopamine-induced hNSCs maintain their phenotype in MPTP-lesioned NHP. Coronal section through the caudate/putamen of an MPTP-lesioned NHP (A), show transplant (TX) of hNSCs counterstained with the nuclear marker DAPI (blue) and immunoprocessed for the human-specific cytoplasmic (purple, hCyt) and the TH (red). (B) Photomicrograph taken from the edge of the graft showing triple labeling with hCyt (purple), TH (red) and the synaptic marker synaptophysin (green). An example of TH+ cell is shown in C, and photomicrograph in D show that the neuritic process extended by the hCyt+ grafted cells (purple) do not express the serotoninergic marker (5 HT, green) indicated by the arrow and expressed by endogenous neurons. The TH+ neurite outgrowth express synaptophysin+ puncta (E). Scale bars are 200 µm (A), 20 µm (B–C), 10 µm (D–E).

**Figure 6:**
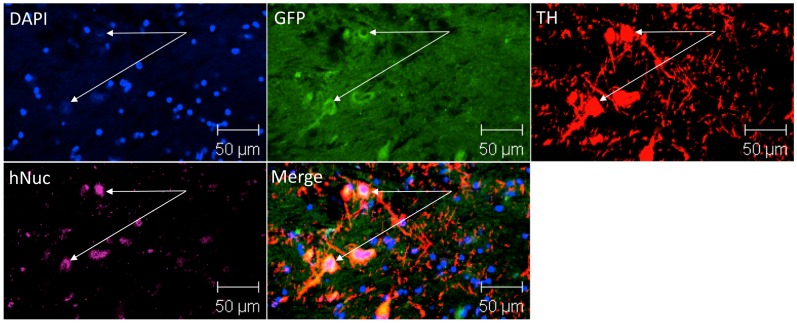
Human NSCs engraft, extend neurites, and differentiate into TH+ neurons in MPTP-lesioned NHPs. Coronal section through the midbain showing grafted DA-differentiated hNSCs into the substantia nigra. Two months-post-transplantation, brains were perfused, sectioned, & immunostained for human-specific & TH markers. Arrows indicate two examples of engrafted donor-derived cells, previously transfected with lenti-GFP, coexpressing the nuclear marker DAPI (bleu), the human nuclear marker hNuc (purple), GFP (green) & TH (red). Scale bar is 50 µm.

To determine the engraftability and stability of the DA-induced phenotype in vivo, hNSCs were cultured under TH-inducing conditions and transplanted into the caudate and substantia nigra of MPTP lesioned NHPs. Two months post-transplantation, the grafted hNSCs were detected with either human-specific nuclei or cytoplasmic markers (Fig. 5A–E) or by the expression of GFP (Fig. 6). The data demonstrated that in all the grafted sites approximately 10% of the hNSCs expressed the dopaminergic phenotype (Fig. 5A,C). These dopaminergic neurons extended neurite outgrowths, co-localized the synaptic marker synaptophysin (Fig. 5B,C,E & Fig. 6) and did not express serotonin (Fig. 5D). Further analysis is necessary to identify the molecular identity of the cells and connectivity at the electron microscopy level. These data suggest that the DA-induced neurons engraft, express dopaminergic phenotype and extend neuritic outgrowth two months post-transplant.

## Discussion

We report the induction of midbrain dopaminergic properties in a self-renewable multipotent hNSC line derived from hESCs. A glial-derived soluble factor acted synergistically with bFGF and LIF to induce the TH expression in these hNSCs. The inductive effect was specific to the DA phenotype, as the GABAergic expression was not altered by the treatment. The evidence that these hESC-derived hNSCs responded similarly as the CNS-derived NSCs [8] to the treatment with the DA instructive signals suggests that these hNSCs are appropriately specified.

The production of a large quantity of DA neurons under defined and reproducible conditions from an unlimited source is necessary for the basic and translational research to develop a safe and efficacious cell therapeutic approach to neurological disorders. Studies have demonstrated that the induction of the dopaminergic phenotype can be achieved in the pluripotent mouse or human ESC cultures enriched for neural progenitors after treatment with SHH and FGF8 [10], [16], [19], [42]. An alternative method based on co-culturing with stromal feeder cells lines (PA6 or MS5) was used to induce or, concomitant with FGF8 and SHH, to maintain the dopamine induction in these enriched neural progenitor cultures [11], [13], [14], [17], [42]–[44]. More recent studies reported the isolation of specific sub-populations of neural precursors from the neural rosette structure based on the cell surface marker Forse1+ expression [38] or on the responsiveness to mitogenic growth factors [39]. These studies suggest that the rosette neural structures contain at least 2 populations of neural precursors with the ability for long-term self-renewal: the Forse1+ with a limited potential of cell proliferation and the EGF+FGF2 responsive precursors that exhibited absence of certain rosette genes, namely *FAM70A, EVI1, ZNF312, LIX1*, or *RSPO3* [39]. The neural progeny we used were isolated from hESCs based on their proliferative response to the mitogenic factors EGF, FGF and LIF [37]. Interestingly, these hNSCs express markers consistent with a midbrain identity and have the ability to self-renew and to differentiate into neurons, astrocytes and oligodendrocytes. Current studies are underway to investigate how each component of the full repertoire of regional identity genes is expressed during the isolation and expansion processes.

We previously demonstrated that bFGF synergizes with the glial-derived factors to instruct stem cell progeny derived from the E14 murine medial and lateral ganglionic eminences to express TH in 10% of the neuronal population [8]. The synergistic action of bFGF and CM turned on the expression of TH gene and transiently increased the expression of the GABAergic phenotype in these forebrain-derived progenitors. In the present study, the hNSCs used express midbrain identity and the treatment with the TH-inducing media did not induce an up-regulation of the GABAergic phenotype. Furthermore, the TH expression did not co-localize with GAD within the same cells. There was a stable low level of GAD expression under control and treated culture conditions suggesting specific actions on the dopaminergic lineage induction. Together these findings suggest that glial CM+GF induces the DA phenotype in the NSC progeny with forebrain or midbrain identity and that the GABAergic co-expression depends on the regional identity of the neuronal progeny.

Two transcription factors, Lmx1a and Msx1, have been reported to be critical for the specification of the midbrain DA neurons [45]. To test the potential application of these findings, Lmx1a and Msx1 were over-expressed in mouse ES cells (mESCs). To induce DA phenotype in mES-derived neural cells, cultures were exposed to bFGF, FGF8 and SHH. In cultures treated with high concentration of SHH (15 ng), the majority of TH-induced neurons co-expressed GABA, whereas Lmx1a over-expressing cultures appeared to limit the TH expression to non-GABAergic cells [45]. In another study, hESC-derived neural progeny treated early with FGF8 reduced the proportion of the TH/GABA expressing cells in neural progenitors that otherwise expressed forebrain transcription factors [16]. Our results showed that the DA induced hNSC progeny engrafted in MPTP lesioned-NHP and differentiated into DA-expressing cells with neurite outgrowth and synaptophysin-expressing terminals. These are encouraging findings and incite us to pursue more elaborated studies of functional effects on parkinsonian deficits.

Our findings suggest that a particular CNS regional identity could be induced in precursors derived from hESCs by a process that is not necessarily the same as the developmentally known differentiation pathways. These different pathways appear to generate the same class of DA neurons; however, they may or may not converge on the same signaling pathways. Recent studies have demonstrated that over-expression of *Ascl1*, *Nurr1* and *Lmx1a* [46] or *Ascl1, Brn2* and *Myt1l* followed by *Lmx1a* and *FoxA2* [47] induced the conversion of fibroblasts to functional dopaminergic neurons. This opens the field to further investigation of the in vivo functionality of these various sources of dopaminergic neurons [48] to ultimately select those that provide functionally effective dopamine replacement in the caudate and putamen.

## Materials and Methods

### Ethics Statement

All animal protocols were approved by the IACUC for Axion Research Foundation/St. Kitts Biomedical Research Foundation. The facility is accredited by AAALAC and all procedures prescribed by AAALAC, OLAW standards, and National Institute of Health (NIH) guidelines were followed including the use of anesthetics and analgesics for surgery and sacrifice and animal welfare conditions for housing and care. Details of animal welfare and steps taken to ameliorate suffering are included in the methods section of the manuscript.

### Cell Cultures

Human neural stem cells (hNSC) were isolated from the H9 and H7 human embryonic stem cell lines (WiCell), as we previously described [37]. The H7 hESC line was genetically engineered to express green fluorescent protein (GFP) to permit additional means to track the grafted cells in vivo. The serum-free culture medium was composed of DMEM/F12 (1∶1) including glucose (0.6%), glutamine (2 mM), sodium bicarbonate (3 mM), and HEPES buffer (5 mM) (all from Sigma, St. Louis, MO, except glutamine from Invitrogen, Carlsbad, CA). A defined hormone mix and salt mixture (Sigma) that included insulin (25 µg/ml), transferrin (100 µg/ml), progesterone (20 nM), putrescine (60 µM), and selenium chloride (30 nM) was used in place of serum. Growth factors including 20 ng/ml epidermal growth factor (EGF), 10 ng/ml bFGF and 10 ng/ml of LIF and conditioned media (CM) were added to the culture two hours after plating. Cells were incubated at 37°C in a 95% air/5% CO_2_ humidified atmosphere.

The differentiation of the hNSCs from passages between 8 and 12 was carried out as follows: hNSCs were spun down and the supernatant was removed and the hNSCs were resuspended in a fresh control media. The hNSCs were single cell dissociated with trypsin and plated under control or TH-inducing conditions (CM+LIF+bFGF) at a density of 5×10^5^cells/ml on poly-L-ornithine-coated (15 mg/ml; Sigma) glass coverslips in 24 well Nunclon culture dishes. [For a step–by-step protocol please see [49]].

#### Growth factors

The growth factors (abbreviation and source) used were: mouse epidermal growth factor (EGF, Upstate Cell Signaling, Lake Placid, NY); human recombinant basic fibroblast growth factor (bFGF) and leukemia inhibitory factor (LIF) (R&D Systems, Minneapolis, MN).

#### Preparation of conditioned media (CM)

CM was prepared from rat glial cell line [8]. The cells were cultured in DMEM/10% FBS until confluence. Confluent glial cell cultures were rinsed once with phosphate buffered saline *(*PBS) and twice with serum-free DMEM/F12 (1∶1) with growth factors and hormone mix and placed in the incubator with 20 ml of the same medium. The CM was collected after 48 hours and centrifuged at 1,000 g and 2,000 g to remove cellular debris. The CM was carefully removed, filtered, aliquoted and stored at −80°C.

### Electrophysisology

Whole-cell voltage-clamp and current-clamp recordings were obtained from visually identified hNSCs at temperature of 30°C. Recordings were made with electrodes (3.0–6.0 MΩ) filled with (in mM): 130 KMeSO_3_, 10 NaCl, 2 MgCl_2,_ 0.16 CaCl_2_, 10 HEPES, 0.5 EGTA. Recordings were performed using a Multiclamp 700 B (Molecular Devices), filtered at 2 kHz and digitized at 10 kHz. For recording spontaneous post-synaptic currents (sEPSCs) and voltage-activated currents, cells were held at −70 mV in voltage-clamp. For evoking action potential, cells were maintained at −70 mV in current-clamp and stimulated with depolarizing current injections. To label recorded cells, biocytin (Invitrogen) was added to all pipette solutions before the experiment with a final concentration of 0.1% and infused to the cell after recording.

### Immunocytochemistry

Mouse monoclonal antibodies and polyclonal antisera raised in different species and directed against neurotransmitter phenotypes and neuronal antigens were used as primary antibodies for indirect immunofluorescence. Polyclonal anti-tyrosine hydroxylase (1∶1000) and anti-dopamine-β-hydroxylase (1∶200) were purchased from Chemicon (Millipore/Chemicon, Temecula, CA). Identical results were obtained with another polyclonal anti-tyrosine hydroxylase obtained from Pel-Freeze Biologicals, Rogers, AR. The following antibodies were also used: anti-DAT (polyclonal 1∶100, Pel-Freeze Biologicals); anti-Nestin (polyclonal 1∶1000, Chemicon); Anti-vimentin (monoclonal 1∶500, Calbiochem, San Diego, CA); anti-Pitx3 (Goat polyclonal, 1∶200, Santa Cruz Biotech); anti-human cytoplasmic marker STEM121 (mouse monoclonal, 1∶100, StemCells Inc.); Anti-3CB2 (monoclonal 1∶500, Developmental Studies Hybridoma Bank, Iowa City, Iowa); anti-β-tubulin class III (monoclonal 1∶1000, Sigma, TuJ1 monoclonal 1∶100 Covance, Berkeley, CA; Polyclonal 1∶200, Aves Labs, Tigard, OR); anti-NeuN (monoclonal 1∶500, Chemicon); anti-glutamic acid decarboxylase (GAD67 1∶1000, Chemicon); polyclonal antibodies against GFAP (monoclonal 1∶1000, Chemicon; polyclonal 1∶200, Aves Labs); monoclonal anti-galactocerebrocide (GC, 1∶200, Chemicon); goat polyclonal anti-FoxA2 (1∶100, Santa Cruz Biotechnology Inc.); rabbit polyclonal anti-Nurr1 (1∶200, Chemicon). Secondary antibodies raised in donkey against mouse, goat and rabbit immunoglobulins, conjugated to the fluorophore rhodamine isothiocyanate (RITC, 1∶200), fluorescein isothiocyanate (FITC, 1∶100) or Alexa Fluor 647 were purchased from Jackson Immunochemicals, West Grove, PA. The same protocol for indirect immunocytochemistry was carried out on cultured cells and on brain tissue sections. Coverslips were fixed with 4% paraformaldehyde for 20 minutes followed by three washes in PBS, 5 min each. Following the PBS rinse, coverslips or tissue sections were processed for dual labeling and incubated with the primary antibodies generated from different species that were added together in PBS/10% normal goat serum/0.3% Triton X-100 for 2 hours at 37°C for coverslips and overnight at 4°C for tissue sections. Following 3 rinses in PBS, secondary antibodies were applied in PBS for 30 min at room temperature for coverslips or 2 hours for tissue sections. Coverslips and tissue sections were then washed three times (5 min each) in PBS, rinsed with water, placed on glass slides, and cover slipped using Fluorsave (Calbiochem) as the mounting medium. Fluorescence was detected, analyzed and photographed with a Zeiss LSM550 laser scanning confocal photomicroscope.

### Morphometric Analysis

The number of TH-immunoreactive (TH-IR) neurons was determined by counting the number of cells at 400× magnification in 15 randomly chosen microscopic observation fields per coverslip. Within the same randomly chosen fields the total numbers of β-III-tubulin-IR neurons and of live cell nuclei stained with DAPI (4′,6-diamidine-2′-phenylindole dihydrochloride) were also determined by examining the surface area of each coverslip at 40× magnification. The total counts were then expressed as percentage of the total DAPI stained nuclei or of the total number of cells expressing the neuronal marker class III β-tubulin. To evaluate the number of grafted human neurons co-expressing TH in brain sections, random sampling of 100 or more cells in the graft regions (core and periphery) was performed at 40×magnification scoring first for hNuc+ or STEM121+, followed by DAPI+ nuclei and then for TH. The double labeling was always confirmed in x-z and y-z cross-sections produced by the orthogonal projections of z-series.

### Quantitative RT-PCR Analysis

Total RNA was extracted from NSCs cultured under different conditions using RNAeasy kit (Quiagen). Aliquots (1 µg) of total RNA from the cells were reverse transcribed (RT) as previously described [8] in the presence of 50 mM Tris-HCl, pH 8.3, 75 mM KCl, 3 mM MgCl2, 10 mM DTT, 0.5 µM dNTPs, and 0.5 µg oligo-dT(12–18) with 200 U Superscript RNase H-Reverse Transcriptase (Invitrogen).

Quantitative real time polymerase chain reaction (Q-PCR), using Applied Biosystems TaqMan Gene Expression Assays was performed in the CFX96 Real-Time PCR Detection System (Bio-Rad) equipped with software for gene expression analysis. The TaqMan Gene Expression Assay ID/Gene Symbol used were: Hs00165941_m1/TH; Hs00158750_m1/LMX1B; Hs00374504_m1/PITX3; Hs00154977_m1/EN1; Hs01105042_m1/DDC; Hs01057416_m1/PAX2; Hs00355914_m1/ALDH2; Hs00428691_m1/NR4A2 (Nurr1); Hs03003631_g1/Eukaryotic 18 S rRNA. The expression of the gene of interest was determined in triplicate for each culture condition. Expression of the reference gene, 18 S, was determined for each sample in triplicate. Quantification was performed at a threshold detection line (“threshold cycles,” Ct value). The Ct of each target gene product was normalized against that of the reference gene 18 S, which was run simultaneously for each marker. Data were expressed as mean ±SEM. The ΔCt for each candidate was calculated as ΔCt of [Ct (target gene) - Ct (18 S)] and the ΔΔCt was the difference between the Ct of treated sample and the Ct of control sample. The relative expression was calculated as the 2^∧ΔΔCt^ according to the methods [50] and plotted as relative levels of gene expression. The amplified products were also run on a 2% agarose Tris-Acetate gel containing 0.5 µg ethidium bromide per ml. The products were visualized through a UV transilluminator, captured in a digital format using Quantify One Gel Analysis software (Bio-Rad Laboratories, Hercules, CA) on a Macintosh G4 computer.

### Methyl-4-phenyl-1,2,3,6-tetrahydropyridine (MPTP) Treatment

The MPTP-monkey model has been previously described and well characterized [51]. Four adult male monkeys (Chlorocebus sabaeus) from St. Kitts, West Indies, were injected intramuscularly with 4 or 5 doses of MPTP HCl (RBI, Natick, MA) given over a 5-day period (cumulative dose 2.25 mg/kg). The MPTP treatment induced parkinsonian symptoms as previously described [51].

### Cell Transplantation

The transplantation technique applied to the MPTP-monkey model has been previously reported [52], [53]. DA-induced hNSCs were single-cell dissociated using trypsin-EDTA in preparation for cell transplantation. Ten µl cell suspensions in PBS at a concentration of 100,000 cells/µl were stereotaxically injected into the caudate and substantia nigra using a controlled perfusion pump at a rate of 1 µl/min, with a 2-minute delay before cannula withdrawal at a rate of 1 µm/min. Following surgery, all animals were clinically observed twice a day for 5 days. Food consumption, body weight, attitude, posture, excreta, appearance and movements were observed and recorded. If an animal experienced loss of appetite, an electrolyte replacement drink, soft foods and fruits were offered. Analgesics were given for 2 days. Antibiotics were administered for 5 days following the surgical procedure. Two months after transplantation, animals were killed by transcardiac perfusion with PBS followed by 4% paraformaldehyde. The brains were cryoprotected in an increasing gradient of sucrose solution (10, 20 and 30%) and cryostat sectioned at 40 µm.

### Immunosuppression

Animals were immunosuppressed by cyclosporine (5 mg/kg/day), prednisone (2 mg/kg tapering to 0.6 mg/kg/day), and azathioprine (starting at 5 mg/kg/day reducing to 1 mg/kg/day) starting 3 days prior to implantation and continuing until sacrifice. To insure effective administration, all doses were administered by injection. Blood levels of cyclosporine were tested periodically by monoclonal specific radioimmunossay by a commercial laboratory (Antech Diagnostics). These doses were well tolerated during the full period of administration.

### Statistical Analysis

Outcome measurements for each experiment were reported as mean±SEM. All data were analyzed using SPSS 11 for Mac OS X (SPSS Inc., Chicago, IL). Significance of inter-group differences was performed by applying Student’s t-test where appropriate. One-Way ANOVA analysis was used to compare group differences. Differences between the means were determined using Bonferroni’s post hoc test. A P-value of less than 0.05 was considered to be statistically significant.
